# Advances in Understanding Lipopolysaccharide-Mediated Hepatitis: Mechanisms and Pathological Features

**DOI:** 10.3390/cimb47020079

**Published:** 2025-01-27

**Authors:** Kazuhiko Nakadate, Hayate Saitoh, Miina Sakaguchi, Fumito Miruno, Naoto Muramatsu, Nozomi Ito, Kanako Tadokoro, Kiyoharu Kawakami

**Affiliations:** Department of Functional Morphology, Meiji Pharmaceutical University, 2-522-1 Noshio, Kiyose, Tokyo 204-8588, Japan; y201111@std.my-pharm.ac.jp (H.S.); y201117@std.my-pharm.ac.jp (M.S.); y201290@std.my-pharm.ac.jp (F.M.); y201295@std.my-pharm.ac.jp (N.M.); s212009@std.my-pharm.ac.jp (N.I.); s212052@std.my-pharm.ac.jp (K.T.); k-kawakami@my-pharm.ac.jp (K.K.)

**Keywords:** lipopolysaccharide, TLR4, hepatitis, pathological features, histopathological diagnosis, electron microscopic observation, CLEM

## Abstract

Lipopolysaccharide (LPS), a key component of Gram-negative bacterial membranes, plays a central role in the pathogenesis of inflammatory liver diseases. In this review, we aimed to explore the role of LPS in hepatic injury. Upon hepatic infiltration, LPS activates Kupffer cells via toll-like receptor 4 (TLR4) signaling, inducing proinflammatory cytokines such as tumor necrosis factor-α and interleukin-1β. These mediators amplify hepatocyte apoptosis, endothelial damage, and platelet aggregation, thereby contributing to sinusoidal thrombosis and tissue ischemia. Pathological features, such as hepatocyte shrinkage, sinusoidal expansion, and fibrin deposition, are hallmark indicators of LPS-induced hepatic inflammation. Therapeutically, aspirin shows promise for attenuating cytokine release, protecting endothelial integrity, and reducing thrombogenesis. Emerging strategies include targeting TLR4 pathways, modulating the gut–liver axis, and utilizing biomolecular approaches such as RNA interference for LPS suppression. The integration of public health interventions, such as dietary optimization and microbiome regulation, offers additional preventive measures. In this review, the dual roles of LPS in inflammation and thrombosis have been emphasized. Advancing our understanding of LPS-driven mechanisms and enhancing treatment strategies are pivotal for managing hepatic inflammation and its systemic implications. Future research should focus on refining biomarkers, optimizing therapeutic efficacy, and addressing safety concerns for clinical applications.

## 1. Introduction

Hepatitis occurs in acute and chronic forms and has significant personal and public health consequences worldwide [1,2]. Acute hepatitis is often caused by short-term exposure to viral infections, toxins, alcohol, or drugs and can lead to acute liver failure and life-threatening conditions [1,3,4]. In contrast, chronic hepatitis progresses through long-term inflammation and can lead to serious organ damage, including cirrhosis and hepatocellular carcinoma [5]. Such liver diseases not only significantly reduce the quality of life of patients but also result in increased medical costs, loss of work capacity, and a growing social and economic burden [6,7]. Therefore, understanding the mechanisms of hepatitis pathogenesis and progression and developing effective treatment strategies based on this understanding have become key challenges in medicine and public health.

The causes of hepatitis are diverse and include viral infections, alcohol ingestion, drug-induced disorders, metabolic abnormalities, and autoimmune diseases [8,9,10]. Recently, lipopolysaccharide (LPS), a cell wall component of Gram-negative bacteria produced by intestinal microbiota, has been shown to play an important role in the development and progression of hepatitis. It acts as a powerful trigger for the immune system, reaching the liver through the portal vein when intestinal permeability and dysbiosis are increased [11,12,13]. The liver is a major organ responsible for detoxification and metabolism and is an immunological “front line” that receives blood directly from the intestinal tract [14]. Therefore, LPS is considered a major factor in the activation of immune cells in the liver and causes inflammatory liver injury [15].

Central to the inflammatory response induced by LPS in the liver is the activation of Kupffer cells (liver-specific macrophages), which produce large amounts of inflammatory cytokines such as tumor necrosis factor (TNF)-α, interleukin (IL)-1β, and IL-6 that trigger an inflammatory cascade in the liver [16,17,18,19]. This process leads to apoptosis and necrosis of hepatocytes, vascular endothelial cell damage, platelet activation, and thrombus formation [20,21,22]. In particular, LPS acts directly on the vascular endothelium of the liver, increasing vascular permeability and causing disrupted blood flow and inadequate oxygen supply, which accelerate the decline in liver function [23].

Furthermore, the LPS-induced inflammatory response has systemic effects that are not limited to the liver. The presence of LPS in the blood promotes the production of C-reactive protein (CRP), an acute phase response protein. CRP is a major biomarker of systemic inflammation, and its elevation suggests effects on other organs in addition to liver inflammation [24,25]. Moreover, platelet activation upon LPS stimulation can lead to progressive thrombus formation, which can cause further tissue damage [26]. Systemic inflammation and vascular damage may also contribute to the progression of chronic and cardiovascular diseases, indicating that LPS has a broad spectrum of effects. Therapeutic strategies are being developed in relation to the inflammatory mechanisms of LPS, and the use of anti-inflammatory drugs, antioxidants, and probiotics has been highlighted as a way to reduce LPS-induced inflammation [27,28,29]. Antiplatelet and anticoagulant agents have also been shown to be useful for preventing thrombus formation [30,31]. In particular, aspirin is considered a promising agent for the treatment of hepatitis and systemic inflammation caused by LPS because of its anti-inflammatory and antithrombotic effects [32,33,34].

Although much research has been conducted on the impact of LPS on liver disease as described above, there are several important research gaps that have yet to be resolved in order to establish a comprehensive treatment and prevention strategy for LPS-induced hepatitis. Filling these gaps is essential to advance the field of research and improve patient outcomes. Among them, it is known that systemic inflammation caused by LPS exacerbates cardiovascular and chronic diseases, but there is a lack of detailed research on specific biomarkers and pathways linking hepatitis to systemic effects. Identification of these could help predict complications and guide appropriate treatment strategies for each patient. To this end, it is first necessary to clarify in detail the mechanisms by which LPS induces hepatitis and thrombosis in the liver and its pathogenesis. Then, the usefulness of currently available drugs and new therapeutic approaches, including public health strategies, will be required.

In this review, we aimed to explore the effects of LPS on the liver and to describe its detailed molecular mechanisms. Moreover, we summarized the pathological diagnostic methods that can lead to therapeutic strategies to control LPS-induced inflammation. These findings are intended to contribute to improving treatments and optimizing public health strategies for patients with liver diseases.

## 2. Materials and Methods

For this review, we used the database contained in PubMed to extract several empirical articles from the relevant literature. Keywords used in the search included “thrombus”, “LPS”, “hepatitis”, “aspirin”, “condition”, and “diagnosis”. In addition, a comprehensive search of the relevant research literature was conducted using PubMed and drug databases for relevance to pathologies used in general practice. We did not use the systematic review and meta-analysis approach in this study.

As for the histological analysis in the present study, we also used the results of the analysis using the technique previously reported by us [33]. That study in animal study was reviewed and approved by the relevant committees of Meiji Pharmaceutical University (No. 2704, 1 April 2023–2024).

## 3. Action of LPS in the Liver

### 3.1. Structure and Immunological Significance of LPS

**Figure 1:**
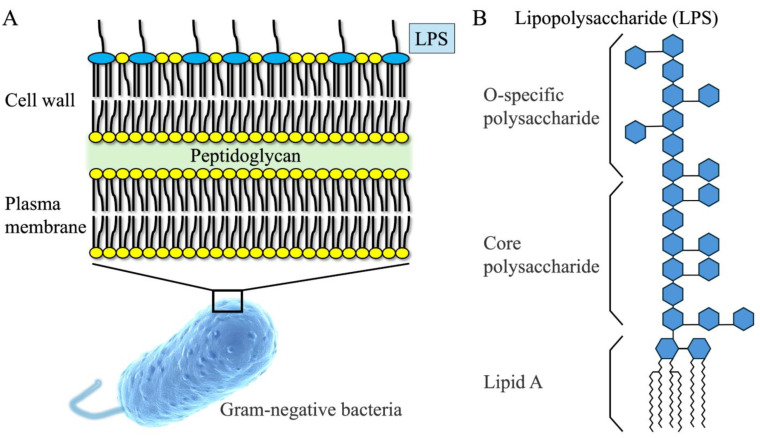
(**A**) Schematic representation of the cell surface structure of Gram-negative bacteria; (**B**) structure of lipopolysaccharide (LPS).

LPS is the major lipid complex that constitutes the outer membrane of Gram-negative bacteria, and its structure and biological functions are closely related to bacterial pathogenicity (Figure 1).

1. Lipid A: Lipid A is the hydrophobic portion of LPS and is at the core of its toxicity and biological activity. Lipid A consists of a disaccharide of glucosamine phosphate attached to a fatty acid that binds directly to toll-like receptor 4 (TLR4) on the cell membrane and triggers an immune response. The composition of fatty acids and the number of phosphate groups vary among bacterial species, contributing to differences in pathogenicity and immune response.
2. Lipooligosaccharide (core polysaccharide): A hydrophilic lipooligosaccharide is the linkage between lipid A and O antigen. This region contains sugar molecules such as keto-oxononanoic acid and heptose, which provide structural stability to LPS.
3. Hydrophilic O-specific polysaccharide (O antigen): This is a long-chain polysaccharide structure located in the outermost layer and is a characteristic region that determines bacterial serotypes. Mutation or loss of the O antigen affects the immune evasion ability of bacteria and is associated with infectivity.

The main mechanism by which LPS induces an immune response is through the activation of signaling pathways via TLR4, a pattern recognition receptor on the cell surface that forms a complex with CD14 and MD-2 to recognize LPS with high affinity [35,36]. This binding triggers intracellular signaling and leads to the activation of nuclear factor kappa-light-chain-enhancer of activated B cells, which promotes the transcription of inflammatory genes [37]. This results in the production of inflammatory cytokines such as TNF-α, IL-1β, and IL-6, leading to acute and chronic inflammatory responses [38,39,40].

In addition, TLR4 signaling functions through MyD88-dependent and TRIF-dependent pathways, which activate distinct pathways. The MyD88-dependent pathway is primarily responsible for early inflammatory responses, whereas the TRIF-dependent pathway induces delayed responses, including interferon-β production. These pathways complement each other and provide the basis for the rapid and sustained response of the immune system to bacterial infections [41,42].

### 3.2. LPS and Inflammatory Response in the Liver

**Figure 2:**
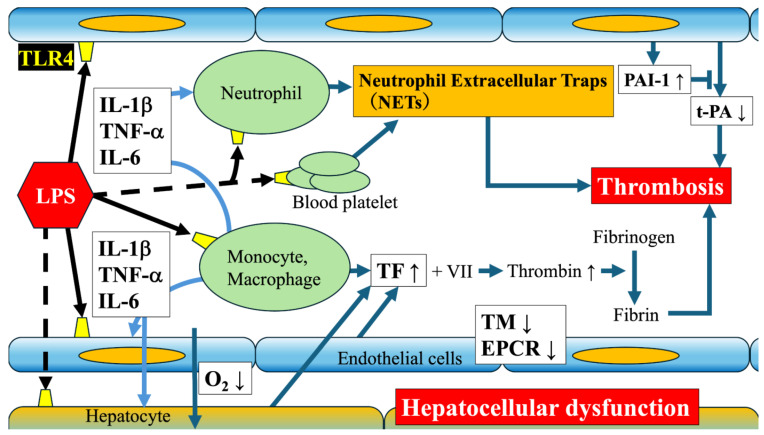
Schematic diagram of intrahepatic sinusoids, various factors induced by LPS, and the mechanism of thrombogenesis. LPS—lipopolysaccharide; TLR4—toll-like receptor 4; IL-1β—interleukin-1β; TNF-α—tumor necrosis factor-α; IL-6—interleukin-6; TF—tissue factor; VII—factor VII of blood coagulation; TM—thrombomodulin; EPCR—endothelial protein C receptor; PAI-1—plasminogen activator inhibitor-1; t-PA—tissue-type plasminogen activator.

The liver receives blood from the intestinal tract via the portal vein and is at the forefront of the LPS derived from intestinal bacteria. As shown in Figure 2, within the liver, cells of the immune system, such as Kupffer cells (liver-specific macrophages) and monocytes, serve as the major LPS response cells [43,44]. Kupffer cells express high levels of TLR4 and CD14 and are capable of rapid LPS recognition and response [45,46].

1. When Kupffer cells recognize LPS, they trigger the following processes:
  - Release of inflammatory cytokines: TNF-α, IL-1β, and IL-6 are secreted and promote recruitment of other immune cells (e.g., neutrophils and lymphocytes) [19,47].
  - Production of chemokines: chemokines such as CCL2 and CXCL1 mobilize immune cells from the surrounding blood vessels to the liver [48].
  - Reactive oxygen species and nitrogen oxide production: Reactive oxygen species (ROS) and nitrogen oxides damage hepatocytes and endothelial cells via oxidative stress [49,50]. Oxidative stress induces hepatocyte death through peroxidation of cell membrane lipids and DNA damage, thereby exacerbating the progression of liver injury [51].
2. Hepatic stellate cells normally function as vitamin A storage cells but are activated by LPS stimulation [52]. This activation promotes the overproduction of collagen and other extracellular matrix components that cause fibrosis. Chronic LPS stimulation may accelerate liver fibrosis that eventually progresses to cirrhosis.
3. Vascular endothelial cells that form sinusoids are directly damaged by LPS, causing a decrease in thrombomodulin and an increase in tissue factor [53,54]. Furthermore, it inhibits tissue-type plasminogen activator by inducing the expression of plasminogen activator inhibitor-1 [55]. This results in the formation of a thrombus. In addition, by inducing changes in vascular permeability, local oxygen supply is restricted and hepatocytes are further damaged [56].

Furthermore, the activation of Kupffer cells produces ROS and nitric oxide, which cause oxidative stress [49]. Oxidative stress induces hepatocyte death through the peroxidation of cell membrane lipids and DNA damage, thereby exacerbating the progression of liver injury [51]. Moreover, LPS activates hepatic stellate cells, which may increase collagen production and promote fibrosis. This is an important pathological change in the progression of chronic liver disease [52].

### 3.3. LPS-Induced Hepatocyte and Sinusoidal Endothelial Cell Changes

LPS-induced inflammation has a wide variety of effects on hepatocytes and endothelial cells, which are the major components of the liver, and leads to various changes in the hepatic microenvironment.

1. Induction of hepatocyte death (apoptosis and necrosis): The release of inflammatory cytokines (especially TNF-α and Fas ligands) by LPS induces apoptosis in hepatocytes [57,58]. Apoptosis is energy-dependent and is characterized by cell shrinkage and nuclear fragmentation; however, at high concentrations of inflammatory cytokines, hepatocytes may enter uncontrolled necrosis [59]. Necrosis leads to further damage to surrounding tissues, forming a vicious cycle that leads to chronic inflammation.
2. Impairment of sinusoidal endothelial cells and blood flow: Sinusoids function as sites of blood circulation and metabolite exchange within the liver, but LPS directly injures sinusoidal endothelial cells [60]. This results in altered vascular permeability and accumulation of inflammatory cells and platelets in the sinusoids. This leads to microthrombus formation and localized blood flow obstruction, resulting in deficient oxygen supply to the liver. Such an impaired blood flow may induce further tissue necrosis and fibrosis.
3. Abnormal platelet aggregation and thrombus formation: LPS activates vascular endothelial cells and platelets and induces platelet aggregation [61,62]. This leads to the formation of microthrombi in portal veins and sinusoids. These thrombi not only interfere with normal blood flow to the liver but also cause additional oxygen deprivation to the surrounding hepatocytes, contributing to chronic damage.

## 4. Diagnosis of Hepatitis

In LPS-induced hepatitis, various blood biochemistry tests are clinically performed. The presence or absence of inflammation and the degree of inflammation can be inferred from the results of these tests, which are useful diagnostic tools. LPS plays an important role in the progression of hepatitis by activating the immune system and stimulating the production of inflammatory mediators. Inflammatory markers, oxidative stress, and chemokine dynamics are indicators of the severity and chronicity of hepatitis. The roles of inflammatory markers and their relevance in LPS-induced hepatitis, including their clinical significance, are discussed below.

### 4.1. Blood Biochemistry Test

Blood biochemistry tests performed in hospitals to diagnose and manage LPS-induced hepatitis are primarily general blood tests that evaluate liver function, inflammation, and coagulation status.

1. Liver function tests:
  - Alanine aminotransferase: specific marker of hepatocyte damage.
  - Aspartate aminotransferase: it reflects damage to the liver and other organs (the heart and muscle).
  - Bilirubin (total bilirubin, direct bilirubin): used to assess bile stasis and liver dysfunction.
  - Albumin: Used to assess hepatic synthetic function. Its levels decrease during chronic inflammation and severe liver injury.
  - Alkaline phosphatase: used to evaluate bile stasis and abnormal bone metabolism.
  - γ-Glutamyltransferase: used to assess liver damage and bile duct disease.
2. Inflammatory markers:
  - CRP level reflects the degree of inflammation and spikes in LPS-induced inflammation.
  - White blood cell count is an indicator of infection and inflammation. Usually, it increases with LPS stimulation.
  - Erythrocyte sedimentation rate is useful to assess chronic inflammation and elevation of LPS-induced hepatitis.
3. Coagulation test:
  - Prothrombin time and international normal ratio: used to assess coagulation capacity and the risk of impaired liver synthesis and disseminated intravascular coagulation.
  - D-dimer: Index of thrombus formation and lysis. It increases during LPS-induced thrombus formation.
  - Platelet count: it is related to thrombus formation and may fluctuate after LPS stimulation.
4. Additional inspection:
  - Abdominal ultrasonography: Used to evaluate liver size, blood flow, and lesions. It is useful for detecting clots and biliary stasis.
  - Blood culture: confirmation of infection by LPS-producing bacteria (e.g., Gram-negative bacteria).
  - Endotoxin measurement: it assesses the amount of LPS (often used in the research phase).

These tests help diagnose and assess the progression of LPS-induced hepatitis and monitor the treatment response. Additional tests may be performed as needed depending on the patient’s condition.

### 4.2. Role and Clinical Significance of Inflammatory Markers

1. C-reactive protein: CRP is widely used as an early indicator of the acute inflammatory response; upon LPS stimulation, CRP is rapidly produced, primarily through IL-6 induction in the liver [63,64]. Elevated blood CRP levels reflect systemic inflammation and indicate the progression of local inflammation in the liver. CRP is an important biomarker for assessing the severity of hepatitis and serves as an early warning for the severity of acute hepatitis and the possibility of sepsis [64].
2. Tumor necrosis factor-α: TNF-α is one of the primary proinflammatory cytokines secreted after LPS stimulation [38,65]. Kupffer cells and macrophages produce TNF-α, which acts on surrounding hepatocytes and endothelial cells to initiate the inflammatory cascade and is involved in increasing vascular permeability, leukocyte infiltration, induction of cell death, and amplification of liver damage. Chronic increases in TNF-α also contribute to the development of liver fibrosis [66].
3. Interleukin-1β: IL-1β is an important proinflammatory cytokine secreted by inflammasomes activated by LPS stimulation [67]. This molecule is a major contributor to the inflammatory environment in the liver and causes damage to the vascular endothelial cells and hepatocytes. In addition, IL-1β induces the production of other cytokines (e.g., IL-6 and IL-8), which contribute to the spread of inflammation [68].

### 4.3. Association Between Increased CRP Levels and Hepatitis Severity After LPS Stimulation

Once LPS enters the bloodstream, an acute phase response is rapidly initiated, and CRP concentrations increase markedly [63,64]. This increase coincides with the peak production of inflammatory mediators and reflects the severity of local inflammation in the liver.

1. CRP as an indicator of hepatitis progression: A rapid increase in CRP levels can be used as a real-time indicator of inflammatory activity. In animal models, CRP concentrations have been observed to increase rapidly within hours of LPS administration and subsequently correlate with inflammatory changes in liver tissue [33]. Therefore, CRP measurement plays an important role in the early diagnosis of LPS-induced hepatitis and monitoring therapeutic efficacy.
2. High CRP levels and risk of chronicity: Persistently high CRP levels suggest uncontrolled inflammation, which increases the risk of hepatitis becoming chronic and transitioning into liver fibrosis. In such cases, the introduction of anti-inflammatory therapy and additional diagnostic procedures is warranted.

### 4.4. Role of Oxidative Stress (Reactive Oxygen Species) and Its Progression to Liver Fibrosis

Oxidative stress is a core pathophysiological process in LPS-induced hepatitis. After LPS stimulation, reactive oxygen species (ROS) production markedly increases, thereby damaging cell membranes, proteins, and DNA. The detailed role of oxidative stress is as follows [69]:

1. Production of ROS and induction of hepatocyte death: Kupffer cells and hepatocytes activated by LPS overproduce ROS, causing hepatocytes to undergo apoptosis or necrosis [16]. This process is closely associated with damage to the mitochondrial membrane and decreased ATP production. Oxidative stress disrupts intercellular signaling pathways and contributes to a vicious cycle of inflammation.
2. Endothelial cell damage and thrombus formation: ROS damage sinusoidal endothelial cells, increase vascular permeability, and induce thrombus formation [70]. Consequently, impaired blood flow and local oxygen deprivation are exacerbated, leading to progressive liver dysfunction.
3. Transition to hepatic fibrosis: Chronic oxidative stress promotes the activation of astrocytes (the major effector cells of intrahepatic fibrosis) [71,72]. Activated astrocytes produce excess extracellular matrix and destroy the normal structure of the liver. This contributes to the development of fibrosis and, eventually, cirrhosis.

### 4.5. Role of Chemokines

Chemokine production after LPS stimulation is important for the mobilization of inflammatory cells and development of liver fibrosis.

1. Types of chemokines and their functions: Chemokines (e.g., MCP-1, CXCL8) are responsible for inducing immune cells such as neutrophils, monocytes, and macrophages to the liver [73,74]. These cells further exacerbate inflammation and damage liver tissue through the production of inflammatory mediators and ROS.
2. Contribution to fibrosis: Chemokines are also involved in astrocyte activation and abnormal signaling between hepatocytes. MCP-1 overexpression promotes the maintenance of chronic inflammation and fibrotic processes [73].

### 4.6. Histopathological Features of LPS-Induced Hepatitis

LPS-induced hepatitis causes significant changes in liver structure and function. These changes can be clearly observed via histological and ultrastructural analyses, allowing a detailed understanding of pathogenesis. Therefore, histopathological features of LPS-induced hepatitis may be described in terms of histological changes and ultrastructural analysis using electron microscopy [33].

#### 4.6.1. Details of Histological Changes

**Figure 3:**
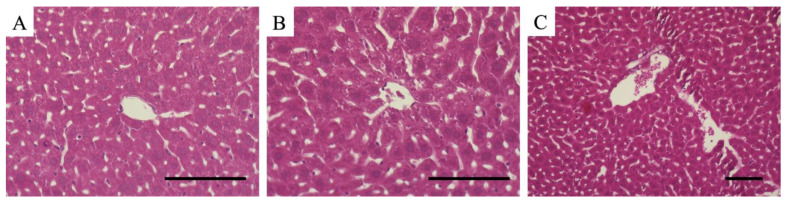
(**A**,**B**) Hematoxylin and eosin-stained images of the liver lobules; (**C**) hematoxylin and eosin-stained image of the intrahepatic vein. (**A**) Normal mouse liver; (**B**,**C**) mouse liver 24 h after LPS administration. As shown in (**A**), the hepatocytes of normal mice are healthy, and staining is even. The sinusoids are normal and few hemocytes are observed in the sinusoids. As shown in (**B**), the hepatocytes in the LPS-treated group are not uniformly stained and are damaged. The sinusoids are swollen. There is an increase in the number of erythrocytes and leukocytes in sinusoids. In (**C**), a large number of blood cells are stored in the vein. All scale bars are 100 μm.

As shown in Figure 3, LPS administration results in multiple pathological abnormalities in the liver tissue. These abnormalities are observed in hepatocytes, vascular structures, and sinusoidal endothelial cells and reflect a progressive inflammatory process.

1. Hepatocyte atrophy and decreased eosin staining: Hepatocyte atrophy is caused by a decrease in cellular metabolic activity and disruption of the cytoskeleton. Trophied cells are characterized by decreased eosin staining. Decreased eosin staining indicates degeneration or degradation of cytoplasmic proteins and reflects the progression of apoptosis (programmed cell death) or necrosis. This phenomenon is closely related to cell death mechanisms associated with the release of inflammatory cytokines (particularly TNF-α and IL-1β).
2. Dilation of sinusoids and increased vascular permeability: Dilatation of the sinusoids indicates a change in hemodynamics. LPS stimulation increases the internal pressure of the sinusoids, resulting in the blockage of blood flow, which is observed in sinusoids. In addition, inflammatory cytokines and ROS damage sinusoidal endothelial cells and increase vascular permeability. This increased permeability allows plasma components and immune cells to leak into surrounding tissues, further expanding the inflammatory response within the liver.
3. Blood cell accumulation and thrombus formation: The accumulation of blood cells in the blood vessels and sinusoids is an important indicator of inflammation progression. This accumulation primarily consists of leukocytes (especially neutrophils) and erythrocytes. These cells form microthrombi by interacting with the endothelial cells and activated platelets. The thrombus causes vascular occlusion, resulting in localized ischemia and an inadequate oxygen supply. This results in accelerated necrosis of hepatocytes and progressive dysfunction of the entire liver.
4. Immune cell infiltration: LPS stimulation causes neutrophils and macrophages to infiltrate liver tissue. These cells release inflammatory mediators and ROS that amplify inflammation and cause tissue damage. In particular, neutrophil overactivation causes direct damage to the surrounding cells and tissues through degranulation.

#### 4.6.2. Details of Scanning Electron Microscope Analysis

**Figure 4:**
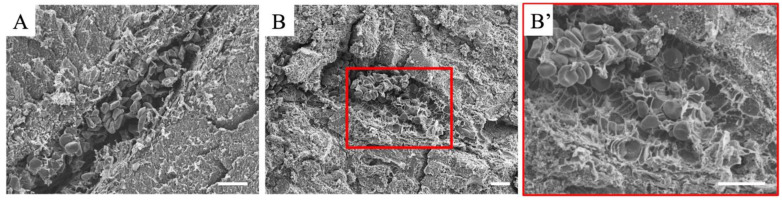
Scanning electron microscopy images of intrahepatic veins 24 h after LPS administration. (**A**,**B**) show different vascular images; (**B′**) magnified image of the red frame in (**B**). A thrombus is observed in the veins. All scale bars are 100 μm.

In addition to optical microscopy, electron microscopy is an extremely important tool for analyzing the ultrastructure of lesions and elucidating the molecular mechanisms of LPS-induced hepatitis. Transmission electron microscopy is useful for detailed intracellular examinations; however, scanning electron microscopy is useful for observing blood cells and thrombi because the sections are extremely thin (Figure 4).

1. Detailed structural observation of blood cell accumulation: Scanning electron microscopy provides a detailed view of leukocyte and red blood cell aggregation in blood vessels and sinusoids. Red blood cell aggregation not only increases the viscosity of the blood stream but also decreases its oxygen-carrying capacity, contributing to systemic hypoxia. Platelets are also observed, suggesting that fibrin formation is an important component of the thrombi.
2. Endothelial cell damage and changes in surface structure: Endothelial cells undergo coarsening of the cell surface under the direct influence of LPS and the action of inflammatory mediators. This coarsening implies the loss of microvilli on the endothelial cell surface and the degradation of intercellular junctions. This results in abnormally increased vascular permeability and accelerated inflammatory progression. In addition, endothelial cells often show signs of apoptosis or necrosis, resulting in the loss of vascular structural integrity.

#### 4.6.3. Details of Transmission Electron Microscope Analysis

Intracellular organelles and cell membrane changes and immunoelectron microscopy can be used to reveal detailed intracellular LPS-induced pathological changes.

1. Changes in intracellular organelles: LPS stimulation of hepatocytes reveals morphological and functional abnormalities in mitochondria. Specifically, mitochondrial swelling, disruption of the cristae structure, and matrix rarefaction are observed. These changes reflect increased oxidative stress and a decreased ability to produce ATP, which promotes cell death. Structural disruption of the Golgi apparatus and rough endoplasmic reticulum also indicates impaired protein synthesis and abnormal stress responses.
2. Localization of inflammatory mediators: Electron microscopy combined with immunogold labeling techniques reveal the localization of inflammatory cytokines (e.g., TNF-α and IL-1β). These cytokines are detected at high concentrations, primarily in Kupffer cells and activated endothelial cells, suggesting that they play a central role in inflammation.

#### 4.6.4. Usefulness of Correlative Light and Electron Microscopy Methods for Pathological Analysis

**Figure 5:**
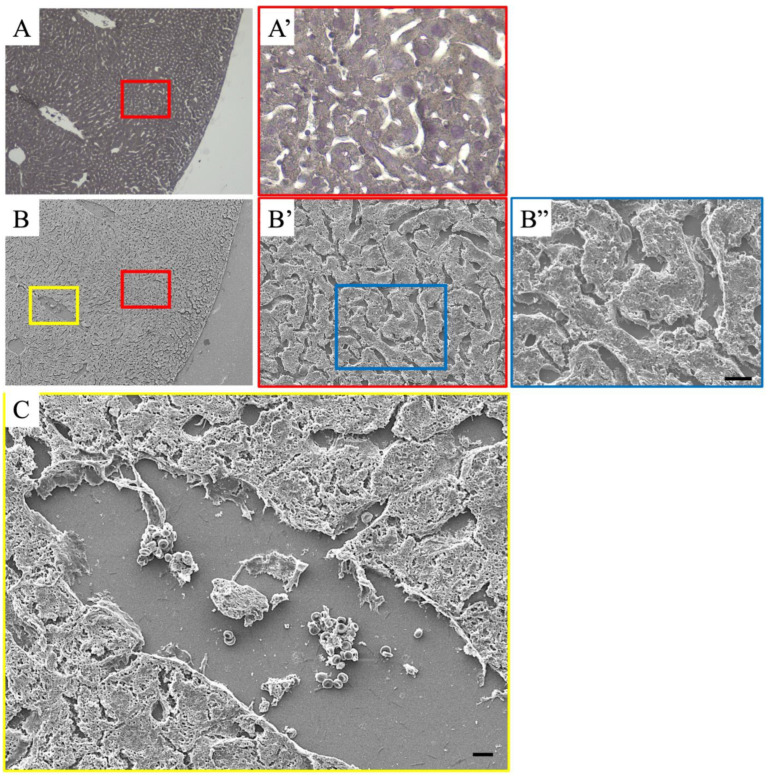
Identical histopathological images obtained using correlative light and electron microscopy. (**A**,**A′**) Hematoxylin-stained images; (**B**–**B″**) scanning electron microscopy images of the same field of view; (**A′**) magnified image of the red frame in (**A**); (**B′**) magnified image of the red frame in (**B**); (**B″**) magnified image of the blue frame in (**B′**); (**C**) magnified image of the yellow frame in (**B**). The morphology of the blood cells is observed using an optical microscope and confirmed using a scanning electron microscope. It is possible to observe blood cells and thrombi in the blood vessels in detail. Scale bar is 10 μm.

Histopathological analysis using optical microscopy is frequently performed in clinical practice. However, analysis using electron microscopy is limited to a small number of cases because of the difficulty and time required to prepare samples. Nevertheless, electron microscopy can be used to prove findings that are difficult to confirm using optical microscopy. Moreover, the correlative light and electron microscopy (CLEM) method, in which the same specimen is analyzed using electron microscopy, may be useful (Figure 5). The CLEM method combines the advantages of optical and electron microscopy to analyze cellular and tissue structures in detail. It is thought to be much less expensive and more effective than conventional electron microscopic analysis and can be used for diagnosis and research. The histopathological analysis of LPS-induced hepatitis has several advantages [33].

1. Structural analysis at high resolution
  - The pattern of inflammation and damage throughout the liver tissue in LPS-induced hepatitis is observed using an optical microscope. Specifically, histological changes such as dilation of sinusoids, accumulation of blood cells, and atrophy of hepatocytes can be observed. Using electron microscopy on the same liver tissue specimen, ultrafine structures (e.g., coarse endothelial cells and morphological changes of platelets and leukocytes) that cannot be observed with optical microscopy can be analyzed in detail.
2. Specific localization analysis at the cellular level
  - The site of inflammation can be identified using optical microscopy, and the morphology and interactions of the cells (Kupffer cells, leukocytes, platelets, etc.) present at the site can be confirmed using scanning electron microscopy.
  - The CLEM method using transmission electron microscopy allows a detailed analysis of LPS-stimulated intracellular structural changes (e.g., lysosomal hypertrophy, nuclear fragmentation, and endothelial cell damage).
3. Detailed analysis of thrombus formation
  - Accumulation of platelets, leukocytes, and red blood cells in the sinusoids can be observed and analyzed to determine their involvement in thrombus formation.
  - Electron microscopy can be used to observe the morphological characteristics of the fibrin network and platelet aggregation that underlies the thrombus.
4. Correlation analysis of pathological changes and molecular functions
  - Detailed analysis of abnormal regions observed via optical and electron microscopy can link pathological changes to abnormalities at the cellular level.
  - The distribution of specific proteins (e.g., tissue factors and inflammatory cytokines) can be confirmed using optical techniques such as immunostaining, and the sites can be further analyzed using electron microscopy.
5. Quantitative analysis
  - By quantifying the number of inflammatory cells, degree of sinusoidal dilation, and frequency of thrombus formation, the progression of LPS-induced hepatitis can be objectively evaluated.
6. Elucidation of complex interactions
  - LPS-stimulated changes in cell–cell adhesion and extracellular matrix can be visualized to reveal the details of inflammation and coagulation processes.

The CLEM method enables multifaceted analysis from the tissue level to the ultrastructural level in LPS-induced hepatitis and contributes to the elucidation of the mechanisms of inflammation and thrombus formation. We believe that this method can provide a basis for the detailed analysis of changes in hepatocytes and endothelial cells and for elucidating new therapeutic targets for inflammatory diseases.

## 5. Therapeutic Approach: Anti-Inflammatory and Antithrombotic

To control the progression of LPS-induced hepatitis, a multifaceted approach that simultaneously suppresses the inflammatory responses and thrombus formation is required. This section focuses on aspirin, a widely used anti-inflammatory and antithrombotic agent, and details its mechanism of action and therapeutic efficacy. In addition, the development status of new treatment methods and the perspective of prevention through lifestyle modification are discussed in depth.

### 5.1. Effects of Aspirin and Its Mechanism of Action

Aspirin is a leading nonsteroidal anti-inflammatory drug, particularly known for its inhibition of the cyclooxygenase (COX) enzyme, which is responsible for prostaglandin synthesis. Prostaglandins play an important role in amplifying inflammatory responses and platelet aggregation [75]. Aspirin inhibits both COX-1 and COX-2 but irreversibly inhibits COX-1, effectively suppressing platelet aggregation and exerting an antithrombotic effect [76]. In contrast, the inhibition of COX-2 suppresses the production of prostaglandin E2 and prostacyclin, resulting in an anti-inflammatory effect that inhibits the secretion of inflammatory cytokines (e.g., TNF-α and IL-1β) [77,78].

Experiments using an LPS-induced hepatitis model have shown that aspirin treatment has a marked effect. Specifically, aspirin treatment significantly reduces the levels of inflammatory markers such as CRP, IL-1β, and TNF-α [33]. These results suggest that aspirin may not only inhibit the progression of inflammation but also prevent the severity of liver injury caused by LPS. Previous studies have reported a decrease in platelet aggregation in the blood vessels and sinusoids, indicating that aspirin has the potential to reduce hepatocyte necrosis and apoptosis through improved blood flow [79,80]. Furthermore, the effect of aspirin is not limited to suppressing inflammatory responses but also involves the control of LPS-induced oxidative stress [81]. Because oxidative stress contributes to hepatocyte death and fibrosis progression, its control is important in the management of LPS-induced hepatitis. Therefore, aspirin is clinically useful as a therapeutic agent with multifaceted actions.

Quantitative data on aspirin therapy have been widely reported in the areas of cardiovascular disease prevention and inflammation control [82,83,84,85]. The data play an important role in the evaluation of therapeutic efficacy and risk of adverse effects. First, many clinical trials have been conducted on the effects of aspirin in cardiovascular disease prevention, with the ASPREE trial being the leading report on primary prevention. This trial examined the effect of aspirin in 19,114 elderly patients and found that the rate of cardiovascular events in the aspirin group was 10.7 events per 10,000 person-years, not significantly different from the placebo group. However, the risk of bleeding was increased in the aspirin group (8.6 events per 10,000 person-years). In secondary prevention, on the other hand, the ISIS-2 trial was conducted in patients with acute myocardial infarction and showed a 23% reduction in vascular death with aspirin (160 mg/day). In the suppression of inflammation, studies have also been conducted on LPS-induced inflammation models. In mouse models, aspirin administration (50–100 mg/kg) has been shown to reduce blood levels of TNF-α and IL-1β by more than 50% and reduce levels of CRP (C-reactive protein) by approximately 30–40% [32,33].

### 5.2. Potential for New Treatment Methods

In the treatment of LPS-induced hepatitis, new therapies are being developed in addition to conventional drugs such as aspirin. Of particular interest are therapies that directly target LPS and its associated signaling pathways. One approach is to regulate LPS-binding proteins (LBPs), which bind to LPS in the blood and are responsible for transferring LPS to TLR4 receptors. Regulation of this process can inhibit TLR4 signaling and prevent excessive inflammatory responses. Therapeutic approaches that utilize soluble TLR4 have also been investigated [86,87]. Soluble TLR4 competes with cell-surface TLR4 for binding to LPS, thus preventing LPS from activating intracellular signaling. This approach has the potential to fundamentally inhibit LPS-induced inflammation.

Additionally, biologics and molecular-targeted therapies are promising. Specifically, monoclonal antibodies that directly inhibit inflammatory cytokines (e.g., TNF-α and IL-1β) and small-molecule drugs that inhibit chemokine signaling have been investigated. These novel therapies are expected to be effective for reducing chronic inflammation and fibrosis in LPS-induced hepatitis.

### 5.3. Perspectives on Lifestyle Improvement and Prevention

Improvement of the intestinal environment plays an important role in the prevention of LPS-induced hepatitis because LPS leaks into the bloodstream owing to an imbalance in the intestinal microbiota or disruption of the intestinal barrier. Regulation of intestinal microbiota can reduce the burden of LPS [88]. Probiotics (lactobacilli and bifidobacteria) and prebiotics (dietary fiber) increase the diversity of intestinal bacteria and decrease intestinal permeability [89,90]. In addition, the intake of zinc and vitamin D, which strengthen the intestinal barrier, may reduce the risk of LPS migration into the bloodstream [91,92].

Nutritional approaches have also attracted considerable attention. Foods containing omega-3 fatty acids and polyphenols contribute to liver health maintenance through their anti-inflammatory and antioxidant effects [93,94]. For example, omega-3 fatty acids inhibit the production of TNF-α and IL-1β, thereby reducing the progression of chronic inflammation [95]. Polyphenols found in green tea and fruits may reduce oxidative stress and prevent LPS-induced hepatocellular damage caused by LPS [96].

## 6. Future Prospects

Although much progress has been made in the study of LPS-induced hepatitis in recent years, several areas remain unexplored. The inflammatory responses induced by LPS and the mechanisms of hepatitis progression caused by these responses are extremely complex, and a deep understanding of these mechanisms is important for future research. In addition, the development of biomarkers, novel therapeutic agents, and public health prevention strategies is essential to establish effective diagnostic and therapeutic strategies. This section details future challenges and prospects for the control of LPS-induced hepatitis.

### 6.1. Further Elucidation of Signaling Pathways in LPS-Induced Inflammation

LPS activates innate immunity via TLR4 and triggers the secretion of inflammatory cytokines (e.g., TNF-α and IL-1β) through various signaling pathways. However, many aspects of these signaling pathways remain unexplored. For example, the crosstalk between MyD88-dependent and MyD88-independent pathways downstream of TLR4 and their involvement in hepatocyte death and fibrosis are not yet fully understood. The roles of LPS-induced oxidative stress and endoplasmic reticulum stress in hepatitis progression require further investigation.

Recent studies have focused on how LPS modulates inflammatory responses through interactions with the gut microbiota. Elucidating how changes in the intestinal environment affect blood LPS levels and the extent to which they contribute as an inflammatory stimulus to the liver may lead to the development of new therapeutic strategies based on the gut–liver axis.

### 6.2. Development of a Predictive Biomarker for LPS-Induced Hepatitis Progression

The development of reliable biomarkers is necessary to accurately assess the progression of LPS-induced hepatitis and determine the timing of appropriate therapeutic interventions. Currently, inflammatory markers such as CRP, IL-1β, and TNF-α are widely used to evaluate hepatitis, but these markers may lack specificity. Therefore, new biomarkers that reflect the hepatocellular damage, thrombus formation, and oxidative stress caused by LPS are needed. Variations in metabolites and microRNAs are potential biomarkers for LPS-induced hepatitis. MicroRNAs play important roles in the regulation of inflammatory responses and cell death and their expression profiles are closely related to the pathophysiology of the liver. Comprehensive proteomic and metabolomic analyses are underway to identify molecular patterns specific to LPS-induced hepatitis.

### 6.3. Evaluation of LPS Inhibitors for Clinical Application

Therapeutic agents that act directly on LPS or target their associated pathways have high potential clinical value. However, cost-effectiveness and safety evaluations are essential for widespread clinical application. Agents targeting soluble TLR4 and LBPs, which are currently under development, are promising new therapeutic tools to reduce LPS-induced inflammation; however, their production costs and side-effect profiles have not yet been fully evaluated.

In particular, the safety aspects require careful consideration of the impact of LPS suppression on the overall immune response. As LPS plays a critical role in the host defense against pathogens, excessive suppression may increase the risk of infection. Therefore, optimizing the dosage and timing of drug administration is a challenge.

### 6.4. Developing Hepatitis Prevention Strategies at the Public Health Level

Public health efforts beyond individual treatments are important to control LPS-induced hepatitis. In particular, lifestyle modifications to prevent hepatitis are an effective way to reduce the inflammatory burden caused by LPS. For example, maintaining balanced intestinal microflora through dietary modifications can reduce blood LPS levels. Because high-fat diets and the excessive intake of refined sugars may worsen the intestinal environment, an active intake of dietary fiber and fermented foods is recommended. In addition, maintaining an exercise routine has been shown to improve intestinal permeability and prevent LPS leakage.

Moreover, it is important to promote early hepatitis diagnosis and appropriate management through health education and screening programs at the community level. It is hoped that by spreading awareness of LPS-related inflammatory diseases, early intervention and preventive measures can be effectively implemented.

### 6.5. Potential Side Effects of Aspirin

Aspirin is widely used as an antiplatelet agent, but its use is associated with several potential side effects and challenges in long-term use. It is important to fully understand these issues and manage them appropriately. The most common side effect is gastrointestinal distress [97,98]. Aspirin is a nonselective inhibitor of cyclooxygenase, resulting in the decreased production of prostaglandins, which are necessary for gastric mucosal protection. This results in an increased risk of gastric ulcers and gastric bleeding, which is of particular concern in the elderly and those infected with H. pylori. It can also cause a range of symptoms from mild abdominal pain to severe gastrointestinal perforation. Next, the risk of bleeding is also a serious issue. Aspirin prolongs hemostasis time by inhibiting thromboxane A2 production and platelet aggregation [99]. This action increases the tendency for systemic bleeding and requires careful management before and after surgery and in trauma patients. Additionally, the risk of cerebral hemorrhage may be increased in hypertensive patients. Other side effects associated with aspirin use include allergic reactions and renal dysfunction [100,101]. In particular, a condition called aspirin asthma can cause a narrowing of the airways and can lead to asthma attacks [102]. Cases have also been reported in which the long-term use of aspirin has decreased renal blood flow and worsened renal dysfunction. Although very rare, hepatotoxicity due to high-dose use is also a concern [103].

Challenges in long-term use include careful identification of indications, thorough management of side effects, monitoring with periodic blood tests, and consideration of alternative therapies if necessary. The use of proton pump inhibitors (PPIs) to prevent gastrointestinal disturbances and at lower doses is recommended. Switching to COX-2 selective inhibitors or other antiplatelet agents (e.g., clopidogrel) is also an option. While aspirin offers significant benefits, the management of side effects is important. It must be used safely and effectively by assessing the risks and benefits for each patient and developing an appropriate treatment plan. In light of these side effects, the effectiveness of thromboprophylaxis in the body’s organs, including the liver, should be monitored.

## 7. Conclusions

LPS is an important virulence factor derived from Gram-negative bacteria and plays a central role in the development of hepatitis. LPS activates the innate immune system and affects the hepatocytes, Kupffer cells, and sinusoidal endothelial cells. This induces the excessive production of inflammatory cytokines, thereby leading to increased oxidative stress, thrombus formation, and fibrosis. These factors accelerate the progression of hepatitis from an acute to a chronic disease.

Although blood biochemical tests are the foundation for the diagnosis of hepatitis, histopathological examinations play an important role. Electron microscopic analysis is particularly useful for observing the localization of inflammatory cells, endothelial cell damage, and detailed structure of thrombus formation. This allows us to elucidate the pathogenesis of LPS-induced hepatitis at the molecular and cellular levels. Aspirin, a nonsteroidal anti-inflammatory drug, has received attention as a treatment option. Aspirin inhibits platelet aggregation by inhibiting COX and reducing the production of inflammatory cytokines. Aspirin has been shown to reduce hepatitis and thrombus formation in LPS-induced hepatitis models. However, owing to the limitations of existing therapies, the development of novel therapeutic agents targeting soluble TLR4 and LBP is required.

Furthermore, lifestyle modifications, such as the adjustment of intestinal microflora and dietary habits, are effective in preventing LPS-induced hepatitis. The intake of fermented foods and dietary fiber improves intestinal permeability and inhibits LPS leakage into the systemic circulation. Early diagnosis and educational activities at the public health level are also key to preventing hepatitis progression. Research on the mechanism of action of LPS and its inhibition strategies has the potential to revolutionize the treatment and prevention of inflammatory liver diseases. Biomarker development and the integration of new diagnostic technologies for personalized medicine are expected to improve the quality of life of patients and public health.

## Data Availability

The datasets used and/or analyzed during this study are available from the corresponding author upon reasonable request.

## Abbreviations

The following abbreviations are used in this manuscript:

LPS	Lipopolysaccharide
TLR4	Toll-like receptor 4
TNF	Tumor necrosis factor
IL	Interleukin
CRP	C-reactive protein
ROS	Reactive oxygen species
CLEM	Correlative light and electron microscopy
COX	Cyclooxygenase
LBP	LPS-binding proteins
